# Genome-Wide Mapping of Cytosine Methylation Revealed Dynamic DNA Methylation Patterns Associated with Sporophyte Development of *Saccharina japonica*

**DOI:** 10.3390/ijms22189877

**Published:** 2021-09-13

**Authors:** Xiaoqi Yang, Xiuliang Wang, Jianting Yao, Delin Duan

**Affiliations:** 1 CAS and Shandong Province Key Laboratory of Experimental Marine Biology, Center for Ocean Mega-Science, Institute of Oceanology, Chinese Academy of Sciences, Qingdao 266071, China; yangxiaoqi@qdio.ac.cn (X.Y.); xlwang@qdio.ac.cn (X.W.); yaojianting@qdio.ac.cn (J.Y.); 2 Laboratory for Marine Biology and Biotechnology, Pilot National Laboratory for Marine Science and Technology (Qingdao), Qingdao 266071, China; 3 University of Chinese Academy of Sciences, Beijing 100049, China

**Keywords:** cytosine methylation, *Saccharina japonica*, sporophyte, development, differentiation, 5-AzaC

## Abstract

Cytosine methylation plays vital roles in regulating gene expression and plant development. However, the function of DNA methylation in the development of macroalgae remains unclear. Through the genome-wide bisulfite sequencing of cytosine methylation in holdfast, stipe and blade, we obtained the complete 5-mC methylation landscape of *Saccharina japonica* sporophyte. Our results revealed that the total DNA methylation level of sporophyte was less than 0.9%, and the content of CHH contexts was dominant. Moreover, the distribution of CHH methylation within the genes exhibited exon-enriched characteristics. Profiling of DNA methylation in three parts revealed the diverse methylation pattern of sporophyte development. These pivotal DMRs were involved in cell motility, cell cycle and cell wall/membrane biogenesis. In comparison with stipe and blade, hypermethylation of mannuronate C5-epimerase in holdfast decreased the transcript abundance, which affected the synthesis of alginate, the key component of cell walls. Additionally, 5-mC modification participated in the regulation of blade and holdfast development by the glutamate content respectively via glutamine synthetase and amidophosphoribosyl transferase, which may act as the epigenetic regulation signal. Overall, our study revealed the global methylation characteristics of the well-defined holdfast, stipe and blade, and provided evidence for epigenetic regulation of sporophyte development in brown macroalgae.

## 1. Introduction

DNA methylation in eukaryotes can be divided into three forms: 5-methylcytosine (5-mC), 6-methyladenine (6-mA), and 7-methylguanine (7-mG) [1,2]. The 5-mC DNA methylation that transfers the methyl group from S-adenosyl-L-methionine to the C5 position of cytosine residues is one of the most conserved epigenetic modifications. It plays important roles in silencing of transposon proliferation [3], the control of genomic imprinting [4], and the regulation of transcript expression [5]. The occurrence of DNA methylation in transposon regions could inhibit the transcription and movement of transposons, protect the genome from uncontrolled insertion, prevent the invasion of foreign DNA, and maintain the stability of chromosomes [6]. In plants, genomic imprinting is independent of de novo DNA methylation [7], and many imprinted genes are regulated by allele-specific activation via DNA demethylase [8]. Additionally, DNA methylation in the promoter region with high tissue-specific expression could influence the transcription patterns by preventing the binding of regulatory proteins [9].

Unlike animals, cytosine methylation in plants occurred mainly at three sequence sites: CG, CHG and CHH (where H represents A, T or C), and de novo establishment of DNA methylation is dependent on the RNA-mediated DNA methylation (RdDM) pathway [5]. Usually, double-stranded RNAs (dsRNAs) are spliced into 24-nt small interfering RNAs by Dicer-like 3 (DCL3), integrated with Argonaute 4 (AGO4) proteins to load into the complex, and recruit domains rearranged methylase 2 (DRM2) to the targeting DNA [10]. After the establishment of de novo DNA methylation, the methyltransferase 1 (MET1) and chromomethylase 2 and 3 (CMT2 and 3) maintain the established methylation status. CMT, the specific DNA methyltransferase in plants, is mainly responsible for the methylation of non-CG sites [11]. The smallest member of the MET gene family in eukaryotic plants, DNMT2, is considered as an evolutionary precursor of DNA methyltransferase, which catalyzes the methylation of both DNA and RNA [12,13,14]. Cooperation division of this methyltransferase contributes to the smooth operation of DNA methylation [15].

In addition to DNA methylation, the 5-mC levels depend on the occurrence of DNA demethylation [16]. Due to the inactivation of DNA methyltransferase, the newly synthesized DNA strand cannot be methylated after DNA replication and passive demethylation occurs. Different from the passive demethylation, the process of active demethylation is dependent on DNA replication, catalyzed by a series of DNA demethylases, such as Repressor of silencing 1 (ROS1), Demeter (DME), Demeter-like 2 (DML2), and Demeter-like 3 (DML3) [17,18]. Recently, a study has found that the DNA demethylases can interact with methylases [19], and such an interaction could provide a potential “bridge” for the negative feedback of dynamic methylation.

Apart from the intrinsic regulation, there are many external factors influencing the DNA methylation levels, such as plant age, tissue development and environment factors [20,21,22]. Generally, the cytosine methylation levels in mature tissues are higher than those in immature tissues [23]. The CHH methylation level is shown to decrease in sperm nuclei, but increase significantly in microspores and sperm [24,25]. All of these reflect that cytosine methylations are undergoing dynamic regulation, which provides a molecular basis for phenotypic plasticity in response to environmental stimulations.

Currently, reports of algal DNA methylation have merely concentrated on a few species, such as *Phaeodactylum tricornutum*, *Chlamydomonas reinhardtii* and *Saccharina japonica*. Approximately 6% of the cytosine in the genome of the marine model diatom *P. tricornutum* is intermittently methylated in a mosaic pattern [26]. The total methylation level is less than 6% in unicellular *C. reinhardtii* and changes of methylation patterns result in significant changes of cell division rates [25,27,28]. Compared with unicellular algae, the methylation level in macroalgae are less reported. Cock et al. found undetectable C5-methyltransferase genes in the genome of macroalgae *Ectocarpus siliculosus*, and this result indicated the lack of DNA methylation in the *Ectocarpales* [29]. Fan et al. reported that the total methylation level of 5-mC in gametophytes of *S. japonica* was only 1.4% and suggested that cytosine methylation plays significant roles in life-cycle stages [30]. However, the role of epigenetic regulation in sporophyte differentiation of macroalgae still remains unknown.

Mature sporophytes of *S. japonica* consist of a holdfast, stipe, and blade of several meters in length, exhibiting simple tissue differentiation characteristics that are different from terrestrial plants and the model algae of *P. tricornutum*, *C. reinhardtii* and *E. siliculosus*. Moreover, the complete genome sequence of *S. japonica* is now available [30], which provides a basis for the single base methylation detection. In this study, we applied the whole genome bisulfite sequencing (WGBS) to explore the cytosine methylation in the holdfast, stipe and blade of sporophytes, and assessed the transcript responses of cytosine methylation loss caused by 5-azacytidine (5-AzaC) treatment. We expected to delineate the complete methylation landscape in the kelp sporophytes, and explore the roles of 5-mC in the sporophyte development, thereby elucidating the epigenetic regulation during kelp growth and development.

## 2. Results

### 2.1. Whole-Genome Bisulfite Sequencing of S. japonica Sporophytes

To describe the whole-genome methylation landscape of *S. japonica* sporophytes, the bisulfite-converted genome DNA fragments from the holdfast, stipe and blade were respectively sequenced by WGBS. The bisulfite conversion efficiencies across the nine samples were higher than 99% (Table 1), ensuring sequencing accuracy. After eliminating raw reads with low quality, duplicate reads, and adapter nulls, the nine sequenced libraries yielded 510.68 million clean reads. The unique mapped reads for each sample covered 85.21–86.05% of the reference kelp genome (Table 1).

### 2.2. DNA Methylation Landscapes in Holdfast, Stipe and Blade

**Figure 1:**
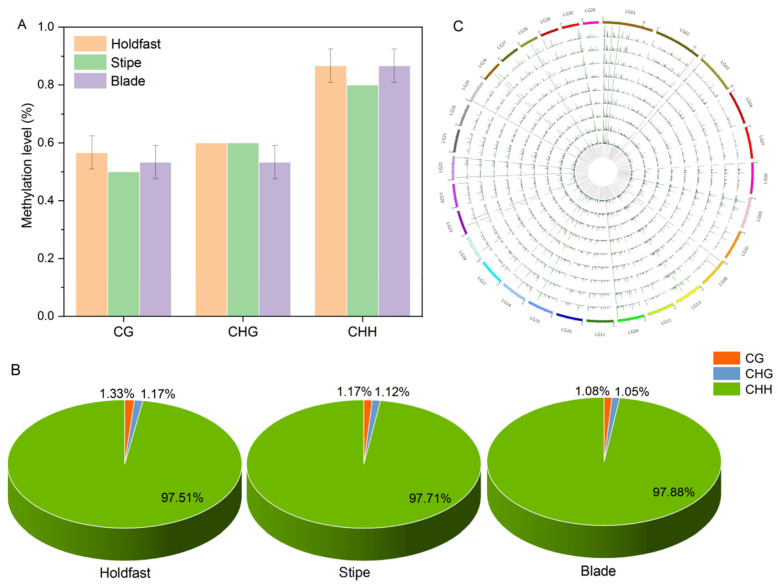
Whole-genome DNA methylation status and chromosomal distribution of *S. japonica* sporophytes. (**A**) DNA methylation levels in holdfast (navajo white), stipe (light green) and blade (medium orchid). (**B**) The relative content of methylated cytosines in CG (dark orange), CHG (cornflower blue) and CHH (lime green) contexts. (**C**) The landscape of DNA methylation in the 31 chromosomes of sporophyte. From outside to inside, the outermost circle is the scale divided according to the corresponding chromosome length. The following nine circles show the genome-wide landscape of DNA methylation in blade (B3–B1), stipe (S3–S1) and holdfast (H3–H1). The innermost circle indicates the gene numbers in the corresponding region. The higher the lines, the higher the methylation levels. B, H and S are the samples of blade, holdfast and stipe, respectively.

To further explore the methylation status at sequence site, the methylated cytosine counts and ratios of CG, CHG and CHH were calculated. By mapping unique mapped reads to the kelp genome with bisulfite conversion, we detected 217.78, 202.97 and 214.51 million cytosines in blade, holdfast and stipe of sporophyte, respectively (Table S1). Of these cytosines, an average ratio of 24.80% cytosines were identified as CG contexts, an average ratio of 19.20% cytosines were identified as CHG context, and an average ratio of 56% cytosines were identified as CHH context (Table S1). At genomic levels, 0.86%, 0.80% and 0.86% of the cytosine sites were methylated in holdfast (H), stipe (S) and blade (B), respectively (Figure 1A). Moreover, the percentage of CHH was the highest in all tissues, followed by CG and CHG sequence (Figure 1A). All of the methylated cytosine in 31 chromosomes of the nine samples were randomly distributed (Figure 1B). Regions with high methylation level were concentrated in Chr 01, and those with low methylation level appeared mainly in Chr 07 and Chr16 (Figure 1C).

**Figure 2:**
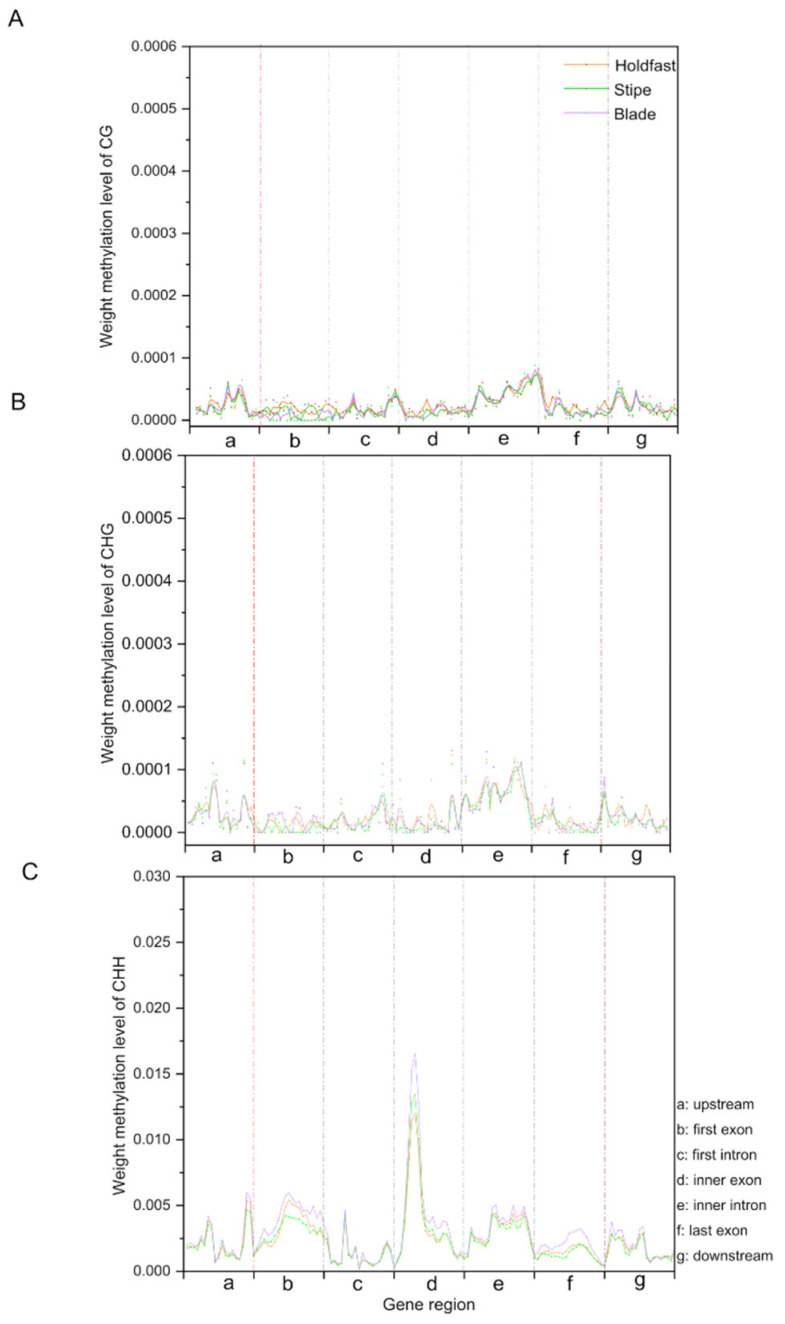
Distribution of DNA methylation levels of CG (**A**), CHG (**B**) and CHH (**C**) context across gene features in holdfast (navajo white), stipe (light green) and blade (medium orchid) of sporophytes. The X axis represents seven genomic features, with a, b, c, d, e, f, and g denoting upstream, first exon, first intron, inner exon, inner intron, last exon, and downstream, respectively. The Y axis indicates the methylation levels of seven gene features.

To compare the genome-wide methylation pattern of various functional genetic elements, we analyzed the methylation status among six different gene regions, including the promoter, 5′UTR (untranslated region), exon, intron, 3′UTR and distal intergenic (Figure 2A–C). Generally, no significant differences between the six gene region methylation patterns were observed in holdfast, stipe and blade, and the methylation levels of presumed functional elements in CG and CHG contexts were less than 0.001. In the CHH context, the highest DNA methylation level was observed in exons, followed by introns, with sites near the transcription terminal site (TTS) showing the lowest methylation level (Figure 2C). At repeat regions, significant enrichment of methylation was observed in repeat bodies, where the CG, CHG and CHH methylation levels were higher in blade than those in holdfast and stipe (Figure S1).

### 2.3. Characterization of DNA Methylation Variations in Holdfast, Stipe and Blade

**Figure 3:**
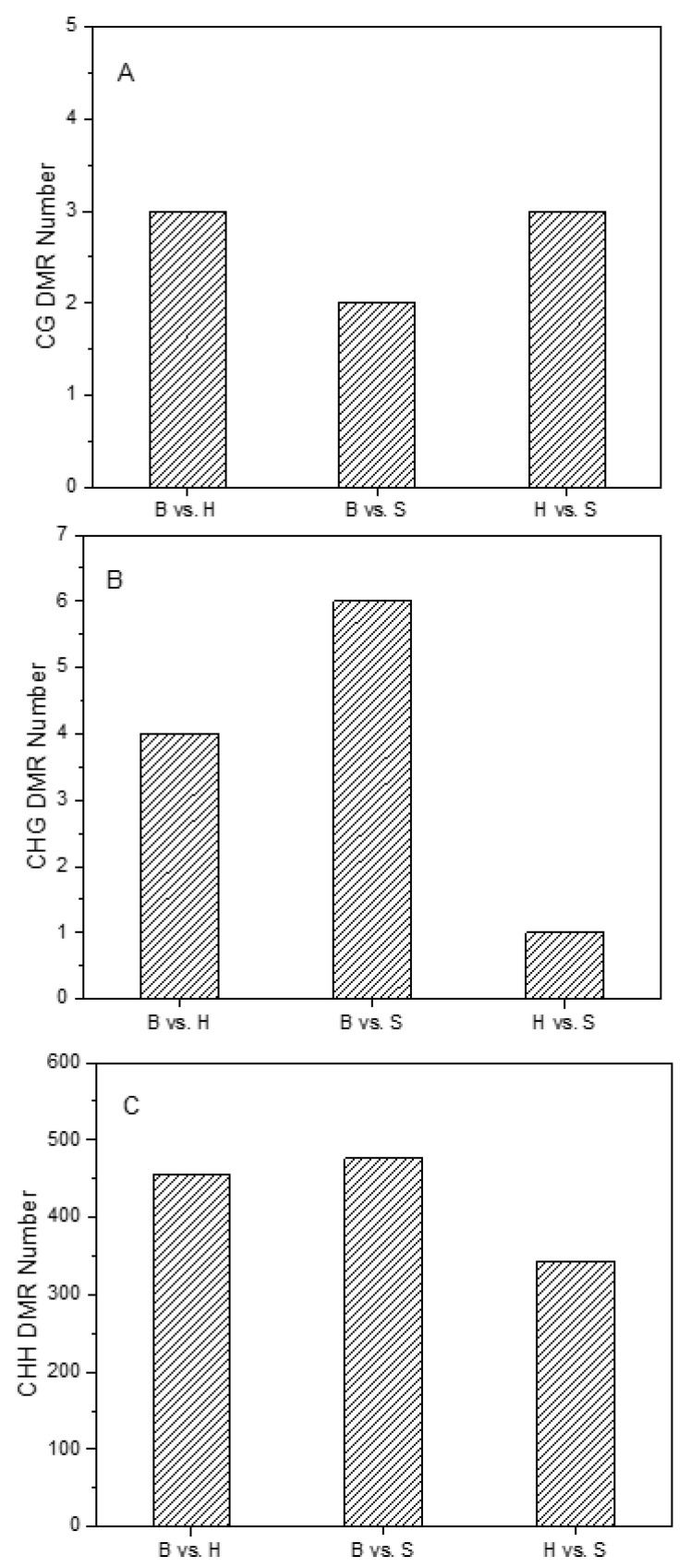
The numbers of detected differentially methylated regions (DMRs) of CG (**A**), CHG (**B**) and CHH (**C**) context in “B vs. H”, “B vs. S” and “H vs. S” groups. B, H and S represented the blade, holdfast and stipe, respectively.

**Figure 4:**
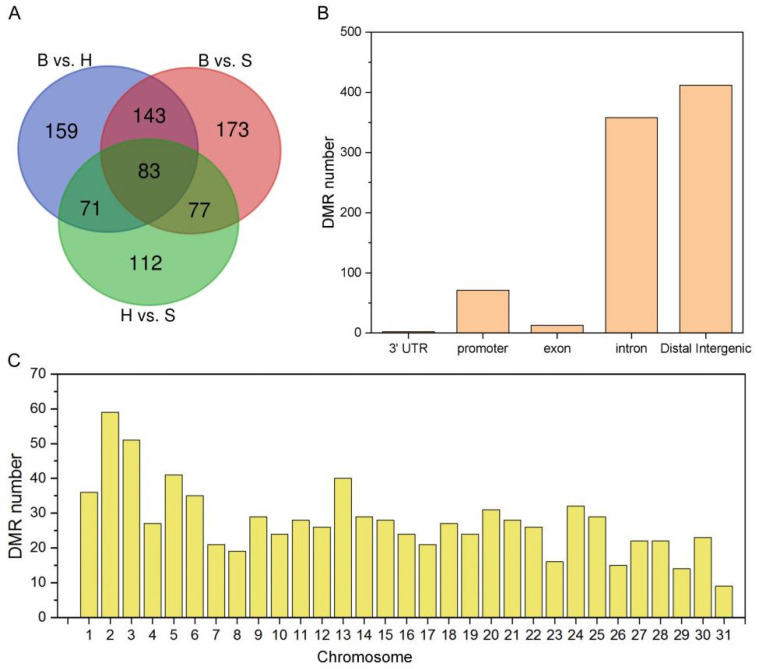
Genomic distribution of DMRs among the holdfast, stipe and blade of sporophytes. (**A**) Venn diagram of DMR numbers identified in “B vs. H”, “B vs. S” and “H vs. S” groups. B, H, and S are the samples of blade, holdfast, and stipe, respectively. (**B**) The distribution of DMRs in different genomic regions, including 3′UTR, promoter, exon, intron and distal intergenic. (**C**) The detailed distribution of DMR numbers in the 31 chromosomes of sporophytes.

To uncover the organ-specific methylation characteristics, we analyzed the differentially methylated regions (DMRs) between holdfast, stipe and blade with at least a 2.0-fold change and a FDR ≤ 0.01. The DMR numbers of both CG and CHG were less than 10 (Figure 3A,B), whereas the DMR number of the CHH context was higher than 360 (Figure 3C). The DMRs of CG and CHG were mainly located in intron and distal intergenic fragments between 0–180 bp (Figure S2A–F). However, for the CHH context, we totally identified 818 DMRs with length range of 0–300 bp (Figure 4A and Figure S2G–I). Approximately 50% of DMRs were located in the 5′UTR, while 40% and 6% of DMRs were located in intron and promoter regions (Figure 4B). Moreover, the DMRs were randomly distributed in the 31 chromosomes without length preferences (Figure 4C).

### 2.4. Annotations of DMR Related Genes in Holdfast, Stipe and Blade

The number of DMR related genes in the group of “H vs. S” was the lowest, indicating similar DNA methylation profiles between holdfast and stipe. The functional annotations of related genes in CG and CHG types are listed in Table 2. Phosphatidylinositol transfer protein SEC14 and related proteins, classified as CG-type related gene, were hypomethylated in both holdfast and stipe (Table 2). The UVR8 receptor in the CHG context was hypermethylated in holdfast when compared to blade (Table 2). For generating the association pathways of DMR related genes in the CHH context, we conducted KEGG enrichment analysis. Top pathways with statistical significance were exhibited in Figure S3. “Glycine, serine and threonine metabolism”, “alanine, aspartate and glutamate metabolism”, “biosynthesis of amino acids” were significantly enriched in groups of “B vs. S”, “B vs. S”, and “H vs. S”, indicating their potential roles in sporophyte development. All of the methylation levels of key associated genes in enriched pathways are listed in Table 3. Additionally, the DMRs that related to “cell motility”, “cell cycle”, “cell wall/membrane biogenesis”, “intracellular trafficking, secretion, and vesicular transport” and “signal transduction” are listed in Table 3. The ankyrin repeat gene that was related to cell wall/membrane biogenesis exhibited hypermethylation in the “B vs. H” group, and exhibited hypomethylation in both “B vs. S” and “H vs. S” groups.

### 2.5. Identification of the DNA Methylation Profiles of Alginate-Related Genes

**Figure 5:**
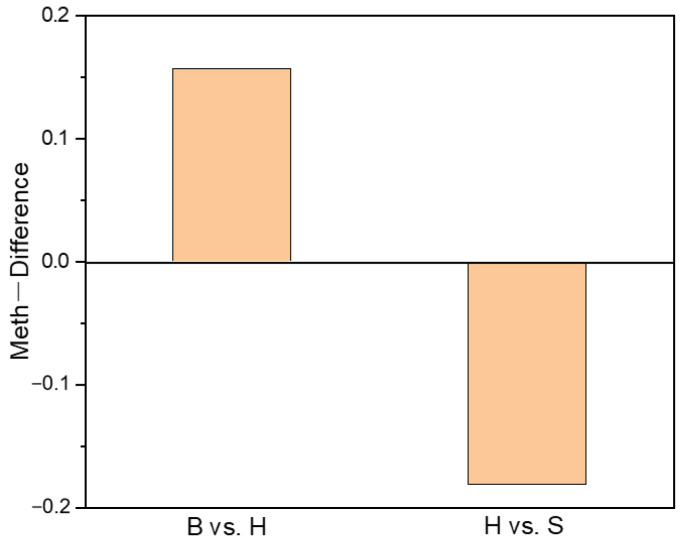
The methylation level of mannuronate C5-epimerase (MC5E) genes (Gene ID: EVM0008148) in the “B vs. H” and “H vs. S” groups. The positive value of methylation level represents the hypermethylation condition, and the negative value of methylation level represents the hypomethylation condition. B, H and S are the samples of blade, holdfast and stipe, respectively.

**Figure 6:**
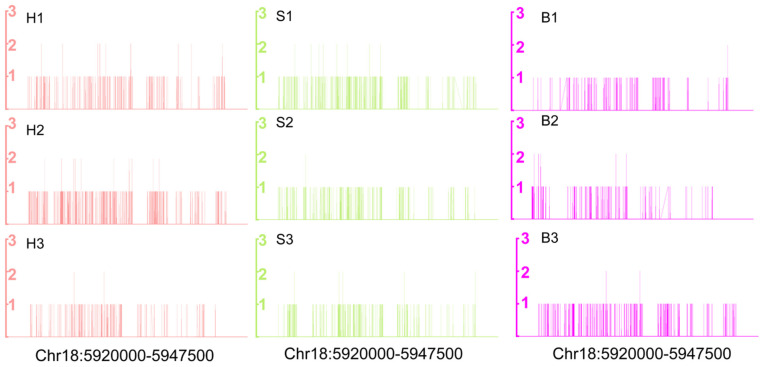
Variation of the DNA methylation pattern of MC5E (EVM0008148) in holdfast (H), stipe (S) and blade (B) through whole genome bisulfite sequencing.

As the raw material for alginate production, alginate synthesis in *S. japoinica* is always the focus of attention. Based on the annotation of the *S. japonica* genome, we screened 48 genes that participate in catalyzing alginate biosynthesis, including 1 mannose-6-phosphate isomerase, 4 phosphomannomutase, 3 GDP-mannose 6-dehydrogenase, 1 beta-1,3-glucan synthases and 39 mannuronate C5-epimerase (MC5E) genes, and identified their three DNA modification patterns of CG, CHG and CHH (Table S2). Among the 48 genes, MC5E (EVM0008148) was hypermethylated in the “B vs. H” group, and hypomethylated in the “H vs. S” group (Figure 5), suggesting a high methylation level in holdfast. DNA methylation profiles of MC5E are shown in Figure 6.

### 2.6. Correlations between the DNA Methylation and Gene Expression

**Figure 7:**
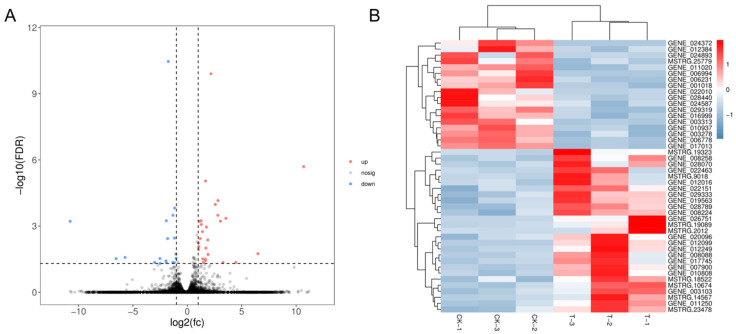
Effects of 5-AzaC treatment on the transcriptome of sporophytes. (**A**) The volcano plot of different expression genes (DEGs) present, in which blue dots indicate down-regulated genes and red dots indicate up-regulated genes in response to 5-AzaC treatment. (**B**) Cluster analysis of differential expression genes. Each row represents one DEG, and the color gradually changes from blue to red, indicating the shift of gene expression level from low to high. CK represents the control group, and T represents the 5-AzaC treatment group.

To identify the regulation of 5-methylcytosine on gene expression, we compared the transcriptomes in the absence (CK) and presence (T) of the 75 µm DNA methylation inhibitor 5-AzaC, which can decrease the DNA methylation level by 20% (Figure S4). RNA-seq analysis showed that 28,164 genes were identified and the genomic mapping ratios were greater than 88% (Table S3). In comparison with the CK group, 27 differential expression genes (DEGs) were up-regulated and 18 DEGs were down-regulated in the T group (Figure 7A,B). Among these up-regulated DEGs, transcriptions of *tyrosine kinase specific for activated (GTP-bound) p21cdc42Hs* (*TR*), “*imm up regulated 3*” (*imm3*), and “*GDSL-like lipolytic enzyme*” associated with cell division and development were included (Table S4). 5-AzaC treatment also resulted in the up-regulation of *mannuronan C-5-epimerase* (*MC5E*). Moreover, the key components of alginate synthesis exhibited an up-regulation tendency, indicating the elevation of alginate synthesis (Figure S5). Among the down-regulated DEGs, transcriptions of “*retrovirus-related Pol polyprotein LINE-1*” and “*choline dehydrogenase*” were down-regulated by −10.76 and −5.71 fold (Table S5). Additionally, eight genes that included both up-regulated and down-regulated genes were randomly selected for verification with quantitative real-time PCR (qRT-PCR) assay, and their expression patterns detected with RNA-seq and qRT-PCR were consistent (Figure S6), indicating the data reliability of RNA-seq.

**Figure 8:**
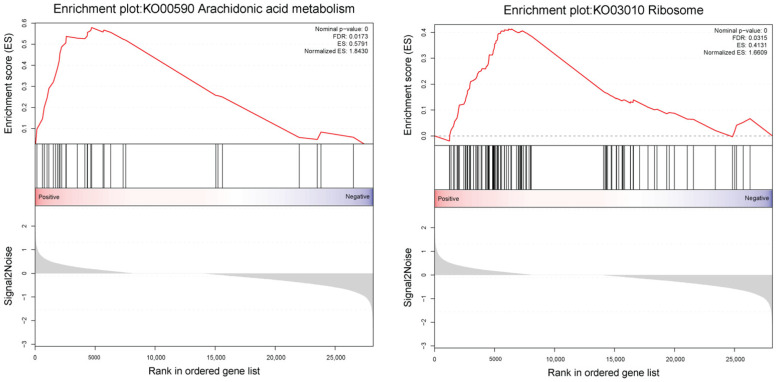
Enrichment plots from the gene set enrichment analysis of KEGG pathways in response to the 5-AzaC treatment of *S. japonica (p*-val. < 0.05). ES indicates the enrich score, and NES indicates the normalized enrich score.

Gene set enrichment analysis (GSEA) showed 7 gene sets were up-regulated in the presence of 5-AzaC, including ribosome, arachidonic acid metabolism, folate biosynthesis, butanoate metabolism, valine, leucine and isoleucin degradation, sulfur metabolism and oxidative phosphorylation (Table 4). Among the 7 up-regulated gene sets, two KEGG-derived gene sets of ribosome and arachidonic acid metabolism were detected in the top 2 gene sets with a *p*-value < 0.05 and a FDR < 0.25 (Figure 8, Table 4).

## 3. Discussion

Referring to the previous high-density SNP-based QTL mapping data, we screened candidate genes related to the blade growth and development of kelp [31], providing a basis for the kelp development analysis. Here in this study, we revealed the cytosine methylation landscape in the holdfast, stipe, and blade of sporophytes at the single-base resolution to elucidate the significance of DNA methylation in the sporophyte development and to understand how the genetic factors determine their development.

### 3.1. Cytosine Methylation Characteristics in the Holdfast, Stipe and Blade

Based on the methylation landscape of holdfast, stipe and blade, the cytosine methylation distribution on 31 chromosomes exhibited stochastic distribution characteristics. In gametophytes of *S. japonica*, the total methylation level of sporophytes was less than 1.4% [30], lower than the unicellular green algae [32]. While in the sporophyte, the total methylation level was only 0.9%, indicating the occurrence of demethylation during the transition from gametophyte to sporophyte. Such DNA methylation changes also revealed the occurrence of genome-wide reprogramming of DNA methylation during the transition of life history of *S. japonica*. Of the three methylation sequences, CHH contexts were dominant for all three parts of the sporophyte. Similar results were reported in the gametophyte of *S. japonica* [30], indicating the dominance of CHH contexts throughout the life-cycle stage. Methylation distributions of the CHH context within the gene regions were enriched in exons, exhibiting ancestral properties of methylome.

### 3.2. Regulations of Cytosine Methylation in the Cell Development of Holdfast, Stipe and Blade

The total methylation level of the holdfast was the highest, followed by the blade, and the stipe was the lowest in the sporophyte. Such a methylation pattern was different in higher plants, which generally exhibited the highest total methylation level in the leaf [25,33], suggesting that macroalgae exhibit different methylation patterns than higher plants. The cytosine methylation regulation in well-defined holdfasts, stipes and blades of sporophytes was further revealed by the numerous DMRs. Moreover, the numbers of DMRs in “B vs. H” and “B vs. S” groups were significantly higher than those in the “H vs. S” group, implying that methylation regulation in the blade was significantly different from both the holdfast and stipe. The pivotal DMRs related to development of sporophytes was the CHH context, which was the most abundant context of DMR.

Generally, collective behaviors of cells are essential for sporophyte development. In this study, we found that the potential DMRs included cell motility, cell cycle and cell wall/membrane biogenesis, suggesting that these cell behaviors were under the regulation of cytosine methylation during sporophyte development. In comparison with the stipe, the ankyrin repeat that played important roles in cell wall/membrane biogenesis was hypermethylated in both the holdfast and blade. Moreover, 5-AzaC treatment resulted in the down-regulation of *Ankyrin Repeat Transient Receptor Potential Channel*, which harbors a large ankyrin repeat domain, disturbing the cell surface receptor signaling. Demethylation of sporophytes significantly up-regulated the transcription of both tyrosine kinase *TR* and *imm 3*. TR is involved in the transport mechanism and the down-regulation of *TR* could inhibit the cellular expansion [34]; *imm 3* is related to the regulation of the sporophyte-specific developmental program and its higher expression may accelerate the development of the meristem [35]. We therefore speculated that the up-regulation of *TR* and *imm 3* caused by the demethylation could promote the cellular expansion and development of sporophytes.

As the main component of cell walls in brown algae [36], the alginate synthesis related genes exhibited the three typical methylation modification patterns in the holdfast, stipe and blade. In comparison with the stipe and blade, the mannuronate C5-epimerase (MC5E) genes exhibited hypermethylation in the holdfast. Moreover, the transcription of *MC5E* was increased after the 5-AzaC treatment. MC5E is involved in alginate biosynthesis to epimerize the M residue to a G residue on polymannuronan and high abundance of *MC5E* ensures a high content of alginate in kelp [35,37]. We therefore speculated that the demethylation in sporophytes could affect the cell wall biogenesis of different sporophyte parts via the regulation of alginate content.

### 3.3. Glutamate May Act as the Epigenetic Regulator in the Sporophyte Development

The enriched pathways of DMRs for all groups concentrated on gene expression and amino acid synthesis, such as the spliceosome and “alanine, aspartate, and glutamate metabolism”, indicating that the DNA methylation mediates the sporophyte development of macroalgae by regulating transcription and amino acid synthesis. Moreover, the up-regulation of arachidonic acid metabolism and ribosome pathways under demethylation treatment confirmed the regulation of DNA methylation on gene expression and amino acid synthesis of sporophytes via affecting transcript abundance. Among the amino acids, the content of glutamate in sporophytes was the highest. Tissue-specific methylation regulation of key genes in glutamate metabolism, including the hypomethylation of amidophosphoribosyl transferase in the holdfast and the hypomethyaltion of glutamine synthetase in the blade, suggested that the DNA methylation modification participated in the regulation of glutamate in the blade and holdfast via different loci. A previous study also revealed that glutamate treatment could increase the DNA methylation level by promoting the expression of DNA methyltransferases [38]. We therefore deduced that glutamate may act as the signal for epigenetic regulation of glutamate content in sporophyte development.

## 4. Materials and Methods

### 4.1. Plant Material and Treatment

Intact sporophytes of *S. japonica* (20–30 cm of length) were collected from the cultivation field in Rongcheng, Shandong province, China, in 2019. After washing with sterile seawater several times to eliminate the epiphytes, the sporophytes were transported to the laboratory and incubated in the dark at 10 °C for 24 h. The holdfast, stipe and blade were cut and then snap-frozen in liquid nitrogen, and were stored at −80 °C for the whole-genome DNA methylation analysis.

Following dark-incubation, the sporophytes were cultured in seawater with 0 or 75 µM 5-AzaC (Sigma, St. Louis, MO, USA) at 10 °C under L/D 10:14. Four days after the treatment, the blades of the sporophyte were collected, frozen in liquid nitrogen, and stored at −80 °C for transcriptome analysis.

### 4.2. Methylation Library Construction and Sequencing Analysis

The DNA of the holdfast, stipe and blade were extracted using the Super Plant Genomic DNA Kit (Polysacchardes & Polyphenolics-rich) (Tiangen, Beijing, China) according to the manufacturer’s recommendations. Following the evaluation of the DNA purity and integrity, 1 µg of qualified genomic DNA was sonicated into sizes between 200–400 bp. The fragmented DNAs were subjected to end-repairing and adenylation. DNA fragments were then treated with bisulfite to convert the unmethylated cytosine into uracil. All the converted fragments were amplified by PCR to construct the final methyl C-seq libraries and then sequenced by an Illumina HiSeq4000 PE101 (Illumina, San Diego, CA, USA) platform.

### 4.3. Bioinformatic Analysis of Methyl C Library Sequencing Data

Raw reads containing low-quality bases, adapter sequences, and undetermined bases were filtered to obtain clean reads. The clean reads were transformed into fully bisulfite-converted (i.e., cytosine-to-thymine and guanine-to-adenine conversions) versions and mapped to the referred genome of *S. japonica* (accession: MEHQ00000000) using bismark software with default parameters. Only the unique best-mapped reads were used to determine the methylation status of cytosine. For each cytosine in the reference genome sequence, the DNA methylation levels were estimated using the ratio of the number of reads supporting mC to the total number of reads. Three contexts of mCG, mCHG, and mCHH methylation levels were analyzed by bismark software in chromosome and gene functional regions (upstream, intron, exon, downstream). The R package was used to construct the chromosome Circos plots of methylation distributions.

### 4.4. Differentially Methylated Regions between the Holdfast, Stipe and Blade

To compare the methylation profiles (mCG, mCHG or mCHH) in the holdfast, stipe and blade, DMRs were identified using MOABS with a threshold of differences of more than 3 cytosines and methylation differences larger than 0.1. The cutoff of methylation analysis was a *p* value < 0.05 using fisher’s exact test to detect the significant DMRs. These reliable mCs were used for further functional analysis. To predict the molecular function of DMR associated genes, Kyoto Encyclopedia of Genes and Genomes (KEGG) pathway enrichment analysis was performed.

### 4.5. cDNA Libraries Construction and Transcriptome Sequencing and Analysis

Total RNAs of samples from control (CK) and 5-AzaC treatment (T) groups were respectively extracted using Trizol reagent kit (Invitrogen, Carlsbad, CA, USA) according to the manufacturer’s protocol. The criteria for high quality RNA included a RNA integrity number > 7.0 from the Agilent 2100 Bioanalyzer (Agilent Technologies, Palo Alto, CA, USA) and a 260/280 spectrophotometric reading > 2.0. Qualified RNA was used for the mRNA enrichment using Oligo (dT) beads. The enriched mRNA was fragmented into short fragments using fragmentation buffer and reverse transcribed into cDNA. Then the cDNA fragments were purified by QiaQuick PCR extraction kit (Qiagen, Venlo, The Netherlands) and ligated to adapters. The ligation products were size selected via 2% agarose gel electrophoresis, PCR amplified, and sequenced using Illumina HiSeq2500. Raw reads obtained from the sequencing was filtered by the removal of adapters and low quality bases to obtain high quality clean reads. Paired-end clean reads were mapped to the reference genome of *S.* *japonica* using HISAT2. 2.4 with default parameters. One fragment per kilobase of transcript per million mapped reads (FPKM) was calculated to quantify the transcript expression abundance and variations by StringTie software (version 1.3.1). Differential expression analysis was performed by DESeq2 (a robust version of edgeR) between CK and T groups. Transcripts with a false discovery rate (FDR) below 0.05 and absolute fold change ≥1 were considered differentially expressed genes. GO enrichment analysis was performed using GSEA, where the cutoff for significance of ES was defined as the score according to a *p* value of 0.05 and an FDR value of 0.25.

### 4.6. qRT-PCR Analysis

Total RNA extraction and preparation was performed by Trizol reagent kit (Invitrogen, Carlsbad, CA, USA). The extracted RNA quality and concentration were examined by agarose gel electrophoresis and DS-11 spectrophotometer (DeNovix, Wilmington, DE, USA). High-purity RNA was reverse transcribed into cDNA using PrimeScript II 1st-strand cDNA synthesis kit (TaKaRa, Dalian, China). PCR reactions were run in an Takara Thermal Cycler DiceTM Real Time System (TaKaRa, Otsu, Japan) using ChamQ SYBR^®^ qPCR Master Mix (Vazyme, Jiangsu, China) according to the directions of the manufacturer. The cycling conditions included an initial incubation at 95 °C for 30 s, followed by 35 cycles of 95 °C for 5 s, and 60 °C for 30 s. The relative abundances of the transcripts were calculated according to the 2^−^^ΔΔCt^ method. β-actin was applied as the internal controls. All the experiments were performed in three biological replicates. Primers used for qRT-PCR are listed in Table S6.

### 4.7. Statistical Analysis

The data were tested by analysis of variance (ANOVA) using SPSS (version 22.0). The *p*-values that were lower than 0.05 were considered to be significant.

## 5. Conclusions

**Figure 9:**
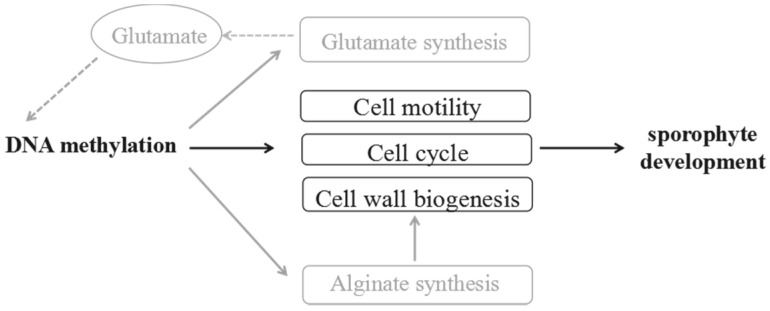
Summary of the effect of DNA methylation on sporophyte development.

Systematic analysis revealed that there were three characteristics of 5-mC modification in sporophytes: (1) the low 5-mC degrees, (2) the dominance of the CHH context and (3) the preference of exons within gene regions. 5 mC methylation participated in the cell development by regulating the algiante synthesis and gluatmate metabolism (Figure 9), mediating the holdfast, stipe and blade development of *S. japonica*. Overall, our studies enhance our knowledge of epigenetic mechanisms during the growth and development of *S. japonica*.

## Figures and Tables

**Table 1:** Summary of the whole-genome bisulfite sequencing data of holdfast (H), stipe (S) and blade (B) in *Saccharina japonica*.

Sample	Number of Clean Reads (Millions)	Unique Beast-Mapped Reads		Bisulfite Conversion Rate (%)
		Number (Millions, % of Clean Reads)	Genome Coverage (%)	
B1	50.87	24.19 (47.55)	83.47	99.45
B2	55.43	27.14 (48.97)	83.54	99.39
B3	59.63	38.22 (64.09)	85.45	99.49
H1	56.79	32.66 (57.51)	85.05	99.4
H2	65.99	38.32 (58.08)	86.05	99.39
H3	56.65	26.36 (46.53)	84.35	99.41
S1	53.99	32.09 (59.43)	85.21	99.44
S2	56.97	28.52 (50.05)	84.03	99.45
S3	54.36	26.67 (49.07)	84.20	99.44
Total	510.68	274.17 (53.48)	-	-

B1, B2 and B3 are the three biological replicates of blade, H1, H2 and H3 are the three biological replicates of holdfast, and S1, S2 and S3 are the three biological replicates of stipe.

**Table 2:** The location and annotation of DMR associated genes of both CG and CHG sites among holdfast (H), stipe (S) and blade (B).

Group	Type	Gene ID	Chr	Region	Annotation
B vs. H	CG	EVM0005705	LG06	Intron	Expressed unknown protein
	CG	EVM0014824	LG16	Intron	Acetyl-CoA acetyltransferase
	CG	EVM0003628	LG17	Distal Intergenic	Phosphatidylinositol transfer protein SEC14 and related proteins
	CHG	EVM0000314	LG11	Distal Intergenic	Ultraviolet-B receptor UVR8
	CHG	EVM0009498	LG24	Intron	Probable tRNA modification GTPase MnmE
	CHG	EVM0013175	LG14	Intron	Conserved unknown protein
	CHG	EVM0015260	LG05	Distal Intergenic	Conserved unknown protein
B vs. S	CG	EVM0007843	LG14	Distal Intergenic	Conserved unknown protein
	CG	EVM0003628	LG17	Distal Intergenic	Phosphatidylinositol transfer protein SEC14 and related proteins
	CHG	EVM0005994	LG20	Intron	Villin
	CHG	EVM0009069	LG28	Intron	Conserved unknown protein
	CHG	EVM0000732	LG11	Distal Intergenic	Conserved unknown protein
	CHG	EVM0010537	LG24	Distal Intergenic	Conserved unknown protein
	CHG	EVM0016237	LG21	Distal Intergenic	Expressed unknown protein
	CHG	EVM0016549	LG11	Promoter	Hypothetical protein Esi00670105
H vs. S	CG	EVM0006317	LG14	Distal Intergenic	Tetratricopeptide repeat containing protein
	CG	EVM0013389	LG25	Distal Intergenic	Conserved unknown protein
	CG	EVM0001259	LG26	Intron	Periplasmic binding protein
	CHG	EVM0010537	LG24	Distal Intergenic	Conserved unknown protein

**Table 3:** The annotations and methylation levels of key DMRs associated genes with holdfast, stipe and blade development.

Pathway	Gene ID	Gene Annotiation	B vs. H	B vs. S	H vs. S
Cell motility	EVM0003386	NB-ARC and TPR repeat-containing protein	●	●	●
	EVM0008642	NB-ARC and TPR repeat-containing protein	●	●	●
	EVM0007683	NB-ARC and TPR repeat-containing protein	●	●	●
	EVM0005188	NB-ARC and TPR repeat-containing protein	●	●	●
Cell cycle	EVM0004313	Cdc2-related protein kinase	●	●	●
	EVM0009233	Asn/thr-rich large protein family protein	●	●	●
Cell wall/membrane biogenesis	EVM0014756	Ankyrin repeat	●	●	●
	EVM0006136	D-isomer specific 2-hydroxyacid dehydrogenase	●	●	●
	EVM0015189	Zinc metalloprotease EGY3	●	●	●
	EVM0015061	Endo-1,3-beta-glucanase, family GH81	●	●	●
Intracellular trafficking, secretion, and vesicular transport	EVM0001662	Glycine dehydrogenase, mitochondrial	●	●	●
	EVM0006566	Plastid Ffh subunit of the signal recognition particle	●	●	●
	EVM0003888	Camkk-meta protein kinase	●	●	●
	EVM0015686	Small Conductance Mechanosensitive Ion channel	●	●	●
	EVM0004673	Uric acid-xanthine permease	●	●	●
	EVM0013751	Protein zinc induced facilitator1	●	●	●
Signal transduction	EVM0004728	Serine/threonine-protein kinase	●	●	●
	EVM0015398	Respiratory burst oxidase homolog protein B	●	●	●
	EVM0004327	Guanylyl cyclase	●	●	●

Pink and blue spots indicated the condition of hypermethylation and hypomethylation, respectively.

**Table 4:** Gene set enrichment analysis of KEGG pathways in response to the 5-AzaC treatment of *S.* *japonica*.

Pathway	Description	ES	NES	*p*-Val.	*q*-Val.
KO00590	Arachidonic acid metabolism	0.58	1.84	0	0.01
KO03010	Ribosome	0.41	1.66	0	0.03
KO00790	Folate biosynthesis	0.41	1.15	0.21	0.49
KO00650	Butanoate metabolism	0.34	0.93	0.55	1.00
KO00280	Valine, leucine and isoleucin degradation	0.26	0.92	0.75	0.85
KO00920	Sulfur metabolism	0.30	0.84	0.72	0.90
KO00190	Oxidative phosphorylation	0.23	0.84	0.85	0.77

ES indicates the enrich score, and NES indicates the normalized enrich score.

## Data Availability

The reference genome of Saccharina Japonica could be retrieved in GenBank at the National Centre for Biotechnology Information with accession number of MEHQ00000000 (https://www.ncbi.nlm.nih.gov/bioproject/?term=MEHQ00000000).

## Supplementary Materials

The following are available online at https://www.mdpi.com/article/10.3390/ijms22189877/s1, Correlation Analysis between DNA Methylation and Gene Expression in Promoter Region. In total, we identified 60 promoter-associated DMR related genes, and randomly selected 26 genes for further transcript analysis. All genes exhibited obvious differential expression in “B vs. H” and “B vs. S” groups (Figure S7). However, negative correlations between the transcriptional abundances and DNA methylation levels were not obvious (Figure S8).
