# Supplementary material for: Genome-Wide Mapping of Cytosine Methylation Revealed Dynamic DNA Methylation Patterns Associated with Sporophyte Development of Saccharina japonica
Source: Int J Mol Sci. 2021 Sep 13;22(18):9877. doi: 10.3390/ijms22189877 (PMC8472486; doi:10.3390/ijms22189877)
Supplement: Supplementary file 1 [file ijms-22-09877-s001.zip › ijms-1345502-supplementary.pdf]

Table S1. Summary of numbers of kelp genome-wide covered cytosines and methylated cytosines.

Sample	Covered cytosines (millions)				Total number of mCs (%)
	Total number of cytosines	Three context types (%)			
		CG	CHG	CHH	
B1	213.70	52.87 (24.74)	41.01 (19.19)	119.82 (56.07)	7.25 (3.39)
B2	212.62	52.67 (24.77)	40.82 (19.20)	119.13 (56.03)	8.56 (4.03)
B3	227.03	56.31 (24.81)	43.57 (19.19)	127.14 (56.00)	10.46 (4.61)
B-Ave	217.78	53.95 (24.77)	41.80 (19.19)	122.03 (56.04)	8.76 (4.01)
H1	197.84	49.15 (24.85)	38.02 (19.22)	110.66 (55.93)	11.01 (5.57)
H2	228.93	56.70 (24.77)	43.90 (19.17)	128.34 (56.06)	12.64 (5.52)
H3	182.13	45.31 (24.87)	35.05 (19.25)	101.77 (55.88)	8.92 (4.90)
H-Ave	202.97	50.39 (24.83)	38.99 (19.21)	113.59 (55.96)	10.86 (5.33)
S1	223.71	55.43 (24.78)	42.91 (19.18)	125.37 (56.04)	9.26 (4.14)
S2	217.99	54.07 (24.80)	41.84 (19.19)	122.09 (56.01)	8.21 (3.77)
S3	201.84	50.01 (24.77)	38.76 (19.21)	113.07 (56.02)	8.83 (4.37)
S-Ave	214.51	53.17 (24.78)	41.17 (19.19)	120.18 (56.02)	8.77 (4.09)

CG, CHG and CHH represent three different cytosine contexts, in which the H represents the A, C or T base. B, H and S are the samples of blade, holdfast and stipe, respectively. Ave represent the mean values.

Table S2. Identification of the DNA methylation modification pattern of alginate synthesis related genes.

GeneID	Symbol	Type	E-value	Identity	Score	Description
EVM0006457	MPI	CG/CHG/CHH	5.45E-93	135/177(76.27)	279	Mannose-6-phosphate isomerase
EVM0002842	PMM	CG/CHG/CHH	5.50E-93	140/141(99.29)	296	Phosphomannose isomerase
EVM0011328	PMM	CG/CHG/CHH	9.61E-152	238/317(75.08)	443	Phosphomannose isomerase
EVM0004835	PMM	CG/CHG/CHH	2.52E-173	251/252(99.60)	520	Phosphomannomutase
EVM0000729	PMM	CG/CHG/CHH	0	295/333(88.59)	588	Phosphomannomutase
EVM0013214	GMD	CG/CHG/CHH	0	320/320(100.00)	662	GDP-mannose 6-dehydrogenase
EVM0002603	GMD	CG/CHG/CHH	0	315/315(100.00)	650	GDP-mannose 6-dehydrogenase
EVM0001282	GMD	CG/CHG/CHH	0	295/314(93.95)	617	GDP-mannose 6-dehydrogenase
EVM0003696	GT2	CG/CHG/CHH	0	448/484(92.56)	930	Beta-1,3-glucan synthases (family GT2)
EVM0013289	MC5E	CG/CHG/CHH	1.98E-75	134/198(67.68)	285	Mannuronan C-5-epimerase
EVM0008148	MC5E	CG/CHG/CHH	0	393/497(79.07)	793	Mannuronan C-5-epimerase
EVM0015347	MC5E	CG/CHG/CHH	0	289/368(78.53)	588	Mannuronan C-5-epimerase
EVM0007897	MC5E	CG/CHG/CHH	3.38E-128	251/336(74.70)	426	Mannuronan C-5-epimerase

EVM0010155	MC5E	CG/CHG/CHH	6.73E-147	227/293(77.47)	428	Mannuronan C-5-epimerase
EVM0004550	MC5E	CG/CHG/CHH	0	361/695(51.94)	632	Mannuronan C-5-epimerase
EVM0005046	MC5E	CG/CHG/CHH	0	330/499(66.13)	676	Mannuronan C-5-epimerase
EVM0006204	MC5E	CG/CHG/CHH	1.12E-101	194/372(52.15)	322	Mannuronan C-5-epimerase
EVM0006486	MC5E	CG/CHG/CHH	2.04E-31	98/236(41.53)	134	Mannuronan C-5-epimerase
EVM0007118	MC5E	CG/CHG/CHH	1.12E-11	62/182(34.07)	73	Mannuronan C-5-epimerase
EVM0007518	MC5E	CG/CHG/CHH	0	361/500(72.20)	746	Mannuronan C-5-epimerase
EVM0008149	MC5E	CG/CHG/CHH	0	293/459(63.83)	572	Mannuronan C-5-epimerase
EVM0008551	MC5E	CG/CHG/CHH	0	356/465(76.56)	706	Mannuronan C-5-epimerase
EVM0009501	MC5E	CG/CHG/CHH	3.44E-94	170/320(53.12)	299	Mannuronan C-5-epimerase
EVM0000842	MC5E	CG/CHG/CHH	0	353/622(56.75)	597	Mannuronan C-5-epimerase
EVM0000944	MC5E	CG/CHG/CHH	0	312/441(70.75)	651	Mannuronan C-5-epimerase
EVM0009520	MC5E	CG/CHG/CHH	7.06E-151	183/198(92.42)	384	Mannuronan C-5-epimerase
EVM0009880	MC5E	CG/CHG/CHH	3.63E-106	186/301(61.79)	353	Mannuronan C-5-epimerase
EVM0009972	MC5E	CG/CHG/CHH	4.96E-109	175/287(60.98)	347	Mannuronan C-5-epimerase
EVM0010857	MC5E	CG/CHG/CHH	9.52E-119	226/460(49.13)	377	Mannuronan C-5-epimerase
EVM0011947	MC5E	CG/CHG/CHH	0	371/496(74.80)	773	Mannuronan C-5-epimerase
EVM0012299	MC5E	CG/CHG/CHH	2.32E-54	123/293(41.98)	195	Mannuronan C-5-epimerase
EVM0012383	MC5E	CG/CHG/CHH	5.18E-123	241/488(49.39)	399	Mannuronan C-5-epimerase
EVM0012837	MC5E	CG/CHG/CHH	5.56E-36	74/114(64.91)	141	Mannuronan C-5-epimerase
EVM0013484	MC5E	CG/CHG/CHH	5.19E-15	41/92(44.57)	79	Mannuronan C-5-epimerase
EVM0014130	MC5E	CG/CHG/CHH	0	320/497(64.39)	664	Mannuronan C-5-epimerase
EVM0001093	MC5E	CG/CHG/CHH	7.53E-173	284/478(59.41)	531	Mannuronan C-5-epimerase
EVM0001223	MC5E	CG/CHG/CHH	6.72E-178	249/319(78.06)	519	Mannuronan C-5-epimerase
EVM0014687	MC5E	CG/CHG/CHH	4.11E-12	72/208(34.62)	76	Mannuronan C-5-epimerase
EVM0014705	MC5E	CG/CHG/CHH	0	428/487(87.89)	888	Mannuronan C-5-epimerase
EVM0014790	MC5E	CG/CHG/CHH	0	310/376(82.45)	641	Mannuronan C-5-epimerase
EVM0016073	MC5E	CG/CHG/CHH	0	363/496(73.19)	734	Mannuronan C-5-epimerase
EVM0016177	MC5E	CG/CHG/CHH	0	397/498(79.72)	827	Mannuronan C-5-epimerase
EVM0016581	MC5E	CG/CHG/CHH	0	367/493(74.44)	747	Mannuronan C-5-epimerase
EVM0002230	MC5E	CG/CHG/CHH	0	385/498(77.31)	807	Mannuronan C-5-epimerase
EVM0002464	MC5E	CG/CHG/CHH	6.08E-27	53/78(67.95)	104	Mannuronan C-5-epimerase
EVM0002592	MC5E	CG/CHG/CHH	4.95E-120	227/452(50.22)	372	Mannuronan C-5-epimerase
EVM0003061	MC5E	CG/CHG/CHH	0	395/497(79.48)	788	Mannuronan C-5-epimerase
EVM0003920	MC5E	CG/CHG/CHH	1.28E-151	257/455(56.48)	473	Mannuronan C-5-epimerase

Table S3. Summary of the output and quality control of *S. japonica* RNA-seq data.

Group	CK	T
Clean Data (bp)	7525209400	8305414400
High Clean Data (bp)	7380026577	8163395445
GC (%)	0.5822	0.5773
Q30 (%)	0.9401	0.940733333
Clean reads	49809940.67	54927310.67
High Clean reads	49385339.33	54014768.67
Total mapped reads	43583266.33	47711446.33
Total mapped ratio	0.882533333	0.8835
Sequenced genes	19895	19653
Sequenced genes ratio	0.7064	0.6978
Total genes	28164	

Table S4. Summary of up-regulated DEGs in response to the 5-AzaC treatment.

No.	Gene ID	log2(FC)	*P*-Value	Description
1	GENE_007900	2.18	<0.01	Ig-like protein
2	GENE_012099	10.69	<0.01	Uncharacterized protein
3	GENE_028789	1.68	<0.01	Tyrosine kinase specific for activated (GTP-bound) p21cdc42Hs/ leucine rich repeat protein
4	GENE_026751	2.82	<0.01	Imm upregulated 3
5	GENE_008258	2.57	<0.01	Unknown
6	GENE_012249	2.81	<0.01	Conserved unknown protein
7	MSTRG.19323	3.55	<0.01	Tyrosine kinase specific for activated (GTP-bound) p21cdc42Hs/ leucine rich repeat protein
8	GENE_029333	1.28	<0.01	Tyrosine kinase specific for activated (GTP-bound) p21cdc42Hs/ leucine rich repeat protein
9	MSTRG.10674	1.12	<0.01	Unknown
10	GENE_008088	3.05	<0.01	Unknown
11	GENE_011250	1.29	<0.01	PKD domain protein
12	MSTRG.19089	1.76	<0.01	EsV-1-166
13	GENE_010808	1.39	<0.01	Conserved secreted protein
14	GENE_028070	1.19	<0.01	GDSL-like lipolytic enzyme
15	GENE_008224	1.88	<0.01	Imm upregulated 3
16	GENE_019563	1.03	<0.01	Mannuronan C-5-epimerase, partial
17	MSTRG.23478	1.01	<0.01	Unknown
18	GENE_022151	1.75	<0.01	Imm upregulated 3
19	GENE_003103	1.08	<0.01	Ig-like protein, group 2
20	MSTRG.2012	6.46	<0.01	Calpain-type cysteine protease DEK1
21	MSTRG.14567	1.91	<0.01	EsV-1-166
22	GENE_020096	1.43	<0.01	PREDICTED: protein LURP-one-related 10-like
23	GENE_017745	1.66	<0.01	EsV-1-166
24	MSTRG.18522	1.68	<0.01	Unknown
25	MSTRG.9018	3.29	<0.01	Vanadium-dependent iodoperoxidase 1
26	GENE_012016	1.49	<0.01	Mannuronan C-5-epimerase C-terminal fragment
27	GENE_022463	4.45	<0.01	Unknown

Table S5. Summary of down-regulated DEGs in response to the 5-AzaC treatment.

No.	Gene ID	log2(FC)	*P*-Value	Description
1	GENE_006778	-1.74	<0.01	Pathogenesis-related protein
2	GENE_029319	-1.16	<0.01	Pheromone shutdown, TraB
3	GENE_017013	-1.32	<0.01	Unknown
4	GENE_022010	-1.93	<0.01	Ankyrin Repeat Transient Receptor Potential Channel
5	GENE_024372	-10.76	<0.01	Retrovirus-related Pol polyprotein LINE-1
6	GENE_016999	-1.18	<0.01	Transporter
7	GENE_006231	-1.80	<0.01	Adhesin-like protein
8	GENE_003313	-1.05	<0.01	ABC transporter
9	GENE_024893	-5.71	<0.01	Choline dehydrogenase
10	GENE_003278	-1.02	<0.01	Hypothetical protein Esi_0045_0060
11	GENE_028440	-2.52	<0.01	Vanadium-dependent iodoperoxidase 1
12	GENE_006994	-6.54	<0.01	Expressed unknown protein
13	GENE_012384	-1.96	<0.01	Unknown

14	GENE_001018	-2.99	<0.01	Unknown
15	GENE_011020	-1.32	<0.01	Carbonic anhydrase
16	GENE_010937	-1.93	<0.01	Polymorphic outer membrane protein
17	GENE_024587	-1.19	<0.01	Unsaturated glucuronyl hydrolase, family GH88
18	MSTRG.25779	-2.49	<0.01	Calcyphosin-like protein

Table S6. Primer used for qRT-PCR. F and R indicate the forward and reverse primers, respectively.

Gene ID	Primer	Primer Sequence	Product length (bp)
GENE_012099	Forward Primer	ACCTTTCCCTAAAGGCCACG	121
	Reverse Primer	CTTTGGTCGGTCGTGGGTTA	
GENE_026751	Forward Primer	ATGTACGCGGTCACGGATAC	251
	Reverse Primer	TTGGCTTGAGCTCTACTGGC	
MSTRG_19323	Forward Primer	TACGGACATCGTCTCCACCT	435
	Reverse Primer	CCGCCCTTACAGGGTTTGAT	
GENE_008088	Forward Primer	CCGACGGGTTTGTGGGATAA	359
	Reverse Primer	CCCCACTGTACCCCATTTCC	
MSTRG_2012	Forward Primer	GAGATCACCACGAACTCCCC	138
	Reverse Primer	CAAACCCTTGTTCGCGAGTC	
GENE_024372	Forward Primer	CGACTCCCGAAACGACTGAT	155
	Reverse Primer	CCGAAGACACGTCTCTGGTC	
GENE_006994	Forward Primer	CGCTCGATGCCAAAGGTTTT	145
	Reverse Primer	CCGGTTAAGACACCCTGACC	
GENE_024893	Forward Primer	GCCGTGGTGGATAAGGACTTCAAG	85
	Reverse Primer	CGGAGGGCGAATAAGGAAAGATGG	
EVM0009376	Forward Primer	GTGGTGGTGGTGGCGTATGTG	114
	Reverse Primer	CCTCGTACTGCTGCTGATGTTCC	
EVM0014652	Forward Primer	GATGCCGCCAACCGATTCAGAG	122
	Reverse Primer	TTCCGCTCCTCCTCCATCGC	
EVM0016386	Forward Primer	GCCGGATAGCCACTAGACAACAC	118
	Reverse Primer	GCCAGCGTTATTCTCCGTCGTC	
EVM0015676	Forward Primer	CTCGTCTTCGTCTTCGTCGTCTTC	106
	Reverse Primer	CGCCGTAACCTTCCGAACCAAG	
EVM0000169	Forward Primer	CTGAGGACGAATCGCTTGACTACG	132
	Reverse Primer	CGGCGGTGTACGAAACTCTTGG	
EVM0008300	Forward Primer	TCACCGCTCAGTCCACCGAAG	81
	Reverse Primer	CGCTCTCCTGCTCGTCATTCAC	
EVM0015316	Forward Primer	AGGAGAGCGGAGACACAATGAGAG	95
	Reverse Primer	TTCTTGGAGAGGCTTGAGCAATGC	
EVM0002335	Forward Primer	CCTCGTCTACCTCCTCATCAGTCC	125
	Reverse Primer	CCACCGTCGCCACCAACAAG	
EVM0016528	Forward Primer	GAGCCAACCACTAACCGCAGAG	149
	Reverse Primer	GCCTTCATCTTGTTTGACGCTTCG	
EVM0000188	Forward Primer	CACAAGAGTTCTACGCCAGGAAGG	82
	Reverse Primer	ACGCCCGAGAGCATGAGGAG	
EVM0002459	Forward Primer	ACGGTCAACATCGACGGTTACAC	142
	Reverse Primer	CATCTCCAAACTCCGTGGCTGAG	
EVM0009861	Forward Primer	CTTCTCGCTGCCTCACGCTTC	85

	Reverse Primer	GCCTCCAATAGCCTTACCACCAAC	
EVM0008139	Forward Primer	CTGCTAGAATCGTCGTTCGGGAAG	123
	Reverse Primer	CAAGTGCTGCGTCTGGTCTCC	
EVM0007683	Forward Primer	TGCCGATTGAAGAAGCCAACCTG	94
	Reverse Primer	CACCGTCCCGCATCTTGAACAC	
EVM0009722	Forward Primer	CCGTCGCAGTCGATCAAGAACC	96
	Reverse Primer	CATGTTGCCGTCGTCGCTCTC	
EVM0014993	Forward Primer	CAACGGCGTGGCTTGGTATCC	82
	Reverse Primer	CAACGGCGTGGCTTGGTATCC	
EVM0014297	Forward Primer	CCACGAGACCATTGAGCAGGATTG	140
	Reverse Primer	CCAGCAAGTCACGGACGAAGC	
EVM0016152	Forward Primer	GCACACTCCCGCTTCGACAC	120
	Reverse Primer	GGCGTACTGTATGCCAACCTCAG	
EVM0014878	Forward Primer	CTTGCCGGTCGAGCCATGAAC	132
	Reverse Primer	CCATGTTCGCCGTGCTGTAGG	
EVM0013496	Forward Primer	CGGGTCCGTGGCGTATTTCG	128
	Reverse Primer	CCACCGTCCACATCGCAATCC	
EVM0009787	Forward Primer	GGTACGGGAGAGGGTCAGCATC	101
	Reverse Primer	GCATGAGCAGCAAGAGGGAGAAG	
EVM0006609	Forward Primer	CTAGCGGCAGCGGGTTTTCC	83
	Reverse Primer	GCGGTTGTGTCTTCCTTCATCTGG	
EVM0007289	Forward Primer	CGATTGTGCTGCACGGAGACC	84
	Reverse Primer	AATCGGCTCTTCTGGACCTCCTC	
EVM0009034	Forward Primer	GAACGCCTCACCACGCTTCG	92
	Reverse Primer	GAACGCCTCACCACGCTTCG	
EVM0011842	Forward Primer	CGGCGAAGACGAGAACGATGAC	88
	Reverse Primer	CCCTGCGAGCCTCCGTAGTAG	
EVM0016415	Forward Primer	GCCGAGGACGCCCATAATGTTC	150
	Reverse Primer	GCTTCGCTGTGGTAGTTGGTGTAG	

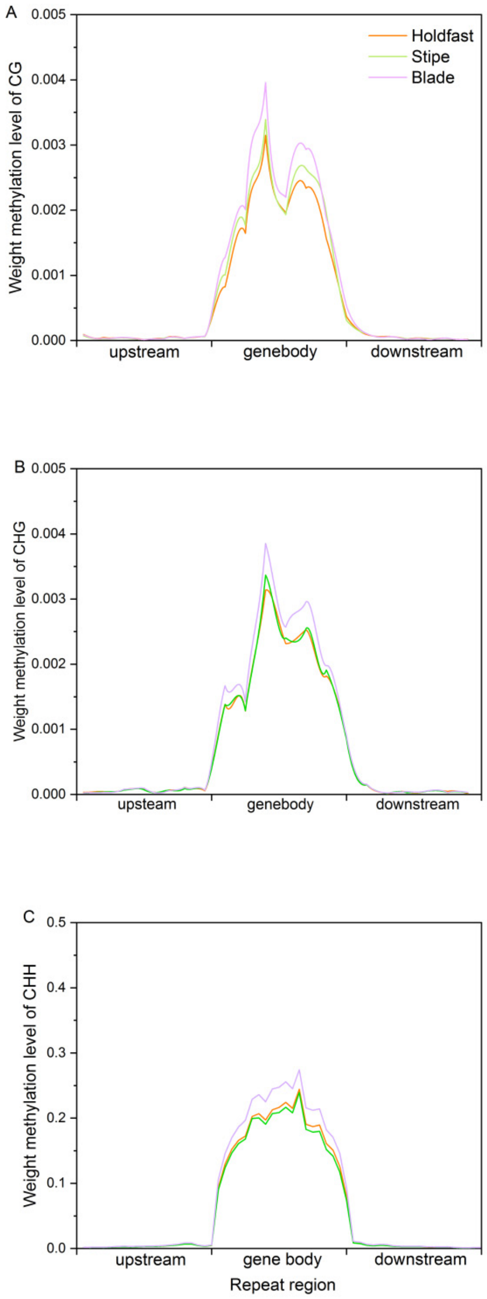

Figure S1. Distribution of DNA methylation levels of CG (A), CHG (B) and CHH (C) context across repeat regions in holdfast (navajo white), stipe (light green) and blade (medium orchid) of sporophyte. Repeat regions include upstream, gene body and downstream. Y axis indicates the methylation levels of seven gene features.

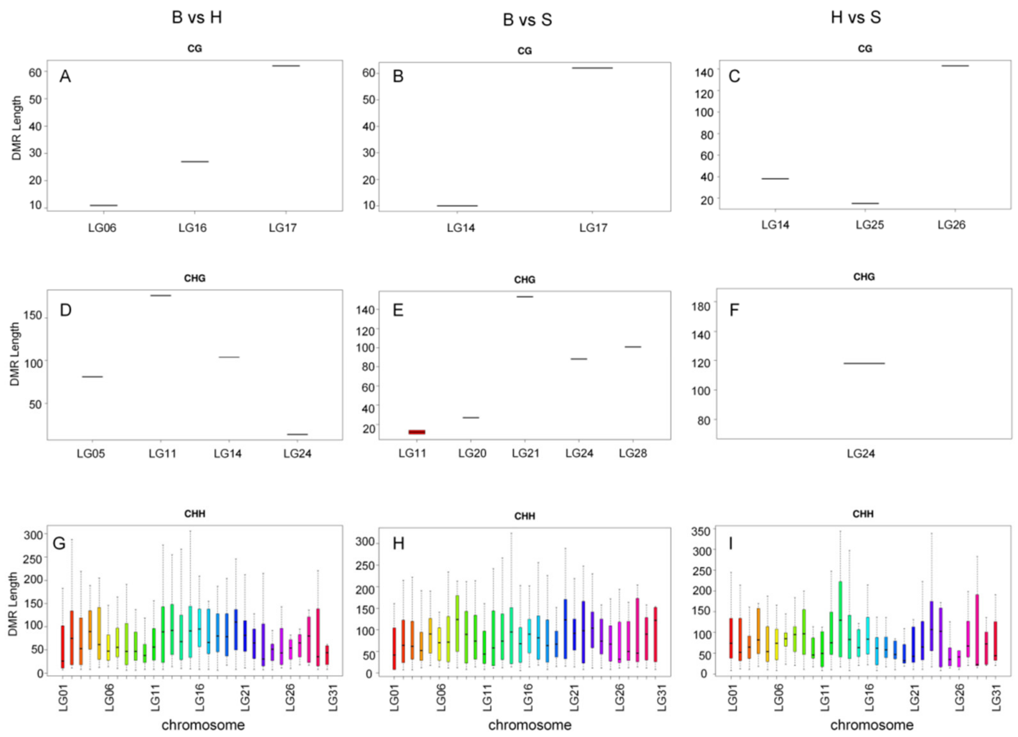

Figure S2. Length distribution of DMRs between the holdfast (H), stipe (S) and blade (B) in *S. japonica* sporophytes. Each row represents a different chromosome, and each column represents a different DMR length.

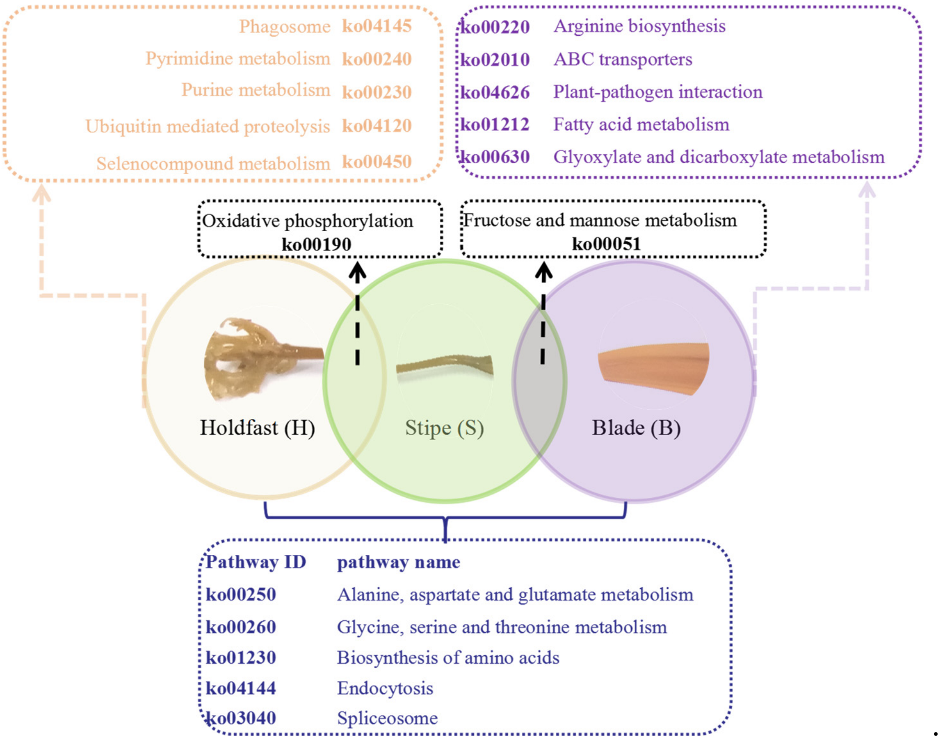

Figure S3. Visualization of enriched pathways among holdfast (H), stipe (S) and blade (B) from DMRs. Blue box includes the pathway ID and name enriched in “B vs. H”, “B vs. S” and “H vs. S” groups, which play important roles in sporophyte development. Navajo white box includes the pathway ID and name enriched in the “B vs. H” and “H vs. S” groups, which play important roles in holdfast development. Medium orchid box includes the pathway ID and name enriched in the “B vs. H” and “B vs. S” groups, which play important role in blade development.

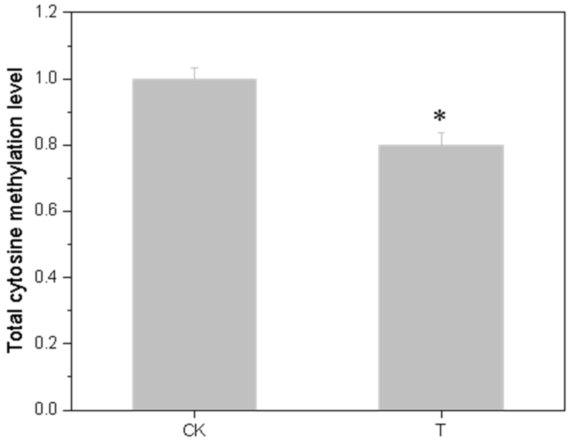

Figure S4. Variation of total methylation level to 5-AzaC treatment in *S. japonica* sporophytes. "CK" and "T" represent control group and 75 μM 5-AzaC treatment group, respectively. * indicated significant difference between "CK" and "T" group.

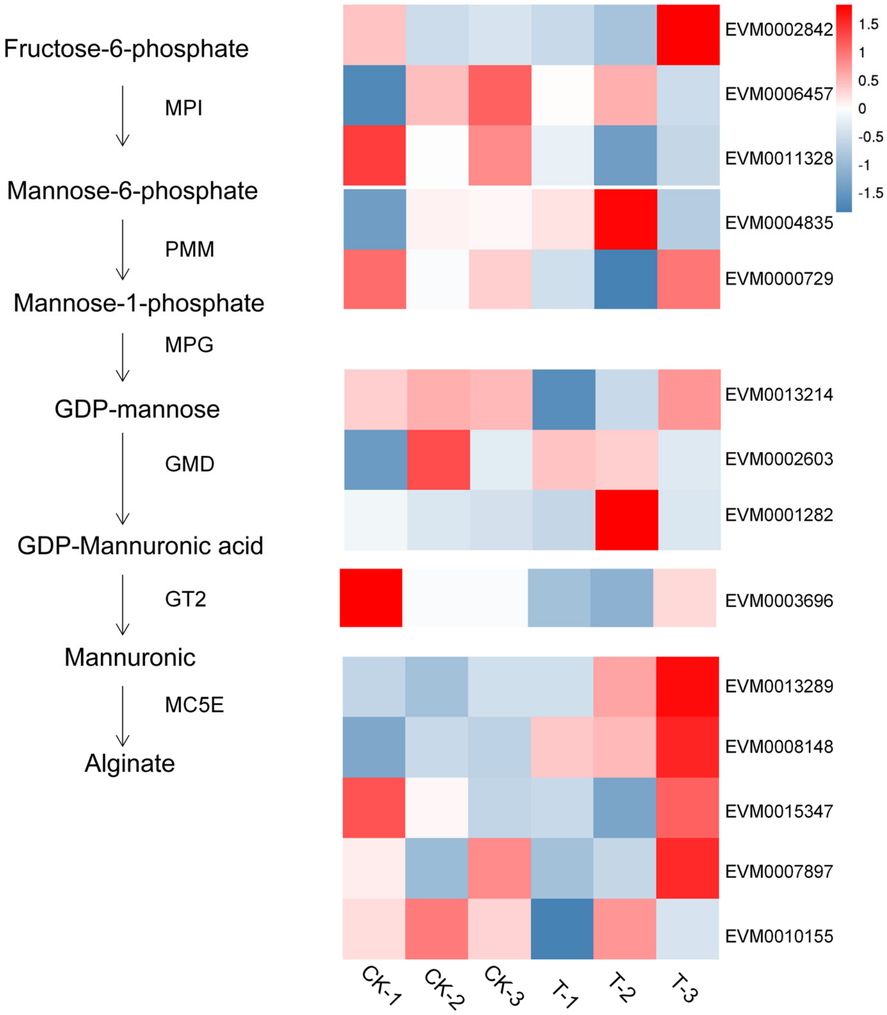

Figure S5. Transcriptional patterns of the genes involved in alginate metabolism of sporophyte in response to 5-AzaC treatment. MPI, Mannose-6-phosphate isomerase; PMM, Phosphomannomutase; GMD, GDP-mannose 6-dehydrogenase; GT2, Beta-1,3-glucan synthases (family GT2); MC5E, Mannuronate C5-epimerase. "CK" and "T" represent control group and 75 μM 5-AzaC treatment group, respectively.

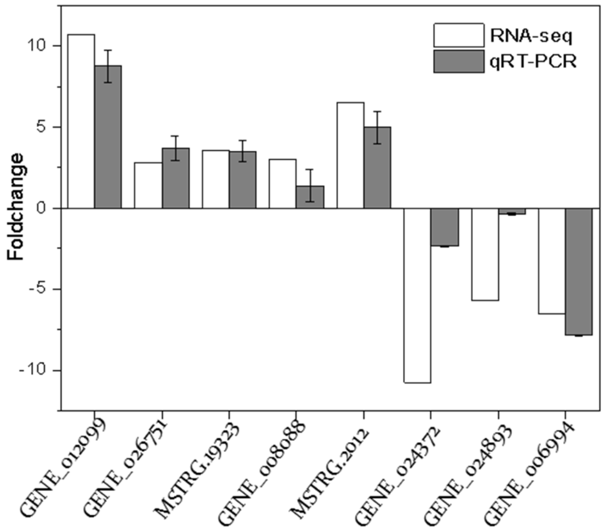

Figure S6. Quantitative real-time PCR (qRT-PCR) analysis of differential expression genes in response to 5-AzaC treatment. The white and dark bars represent the gene expression level measured by RNA-seq and qRT-PCR, respectively.

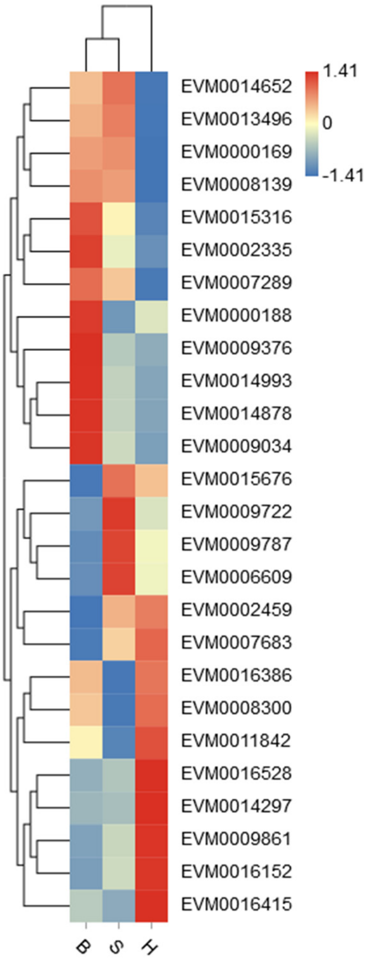

Figure S7. Expression patterns of DMR-related genes that located in promoter region among holdfast (H), stipe (S) and blade (B) of sporophyte. Each row represented one gene, and the color gradually changed from blue to red, indicating the shift of gene expression level from low to high.

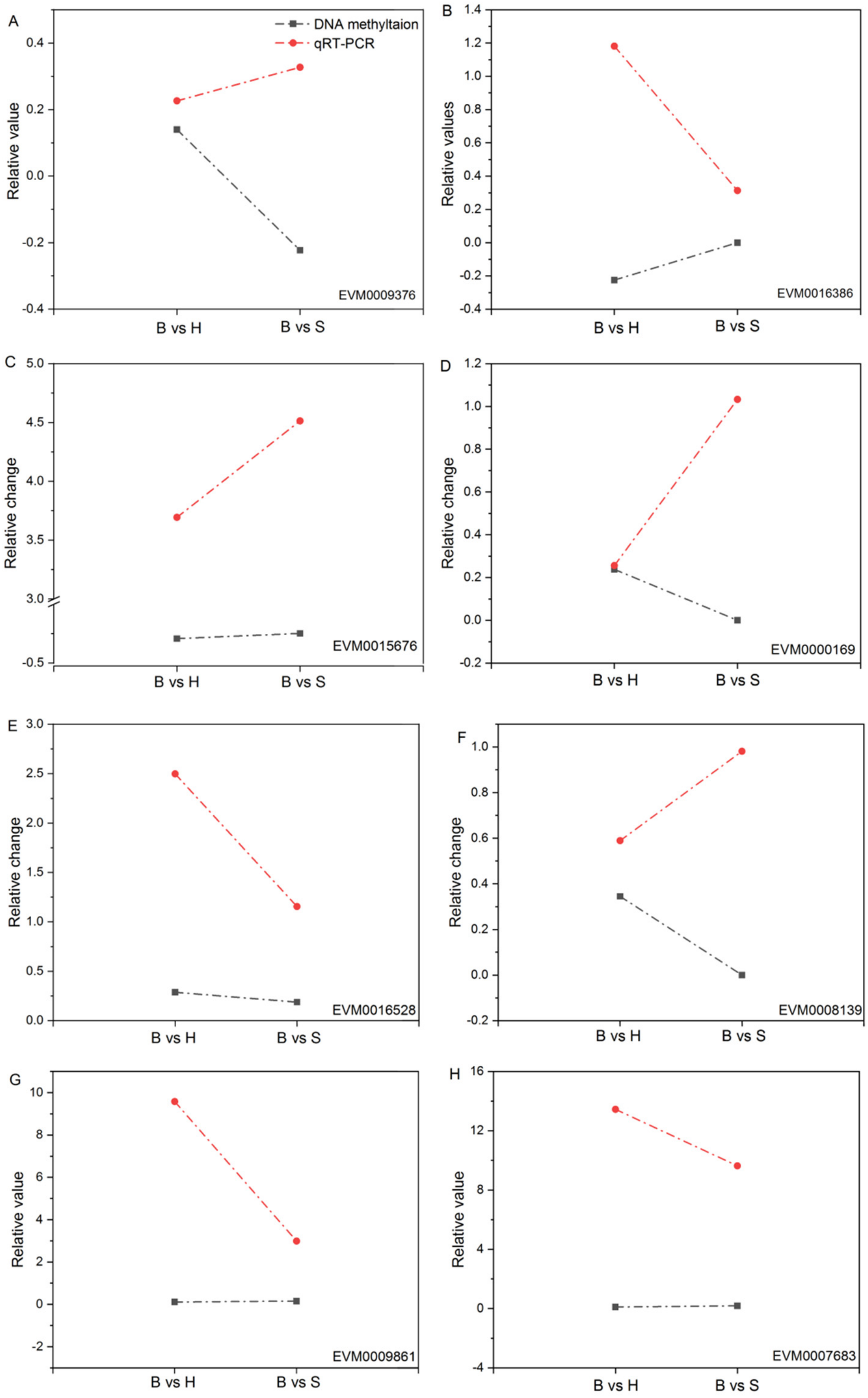

Figure S8. The correlation between DNA methylation level and transcript abundance among the "B vs. H", "B vs. S" groups.
